# A virtual pediatric rheumatology teaching initiative for physicians in Somaliland

**DOI:** 10.1186/s12969-022-00758-8

**Published:** 2023-01-02

**Authors:** Muna Mahfud, Matthew Jones, Tim Fader, Emily Hause

**Affiliations:** 1 Present Address: Pediatric Rheumatology, Allergy, & Immunology, University of Minnesota, Academic Office Building, 2450 Riverside Ave S AO-10, 55454 Minneapolis, MN USA; 2 Director Hope Family Medicine, Amoud University, W6WF+C4F, Amoud Valley, 5 Km East of Borama Borama AD, Boorama, Somalia; 3 Minneapolis, MN USA; 4 Clermont, FL USA

## Letter to the editor

Dear Editors

There is a global shortage of physicians trained in pediatric rheumatology and the African continent is resilient in the face of this shortage [1]. Thanks to collaborations such as the Pediatric Society of the African League Against Rheumatism (PAFLAR), awareness of pediatric rheumatic diseases and the number of pediatric rheumatology trained physicians is growing [2]. However, most African countries, including the Republic of Somaliland, still lack physicians with pediatric rheumatology training. To expand access to pediatric rheumatology knowledge in Somaliland, we designed a virtual educational experience to expose physicians to pediatric rheumatic diseases and treatments.

Faculty and residents from Edna Adan University Hospital in Hargeisa and Amoud University’s family medicine program, Hope Family Medicine, in Borama, participated in four, 30 minutes, interactive, case-based sessions via Zoom. Sessions introduced the pediatric musculoskeletal (MSK) exam tool pediatric Gait, Arms, Legs, and Spine (pGALS) [3], as well as differential diagnoses and treatments for juvenile idiopathic arthritis, juvenile dermatomyositis, systemic lupus erythematosus, and vasculitis. The teaching material from the sessions was sourced from the Pediatric Musculoskeletal Matters (PMM) online material [4] plus published case reports of patients on the African continent.

Prior to the sessions, fewer than half 9/19 (47%) of participants reported having been taught pediatric MSK exam skills whereas 18/19 (94.7%) had been taught adult MSK exam skills. The majority, 12/19 (75%) had no prior exposure to pGALS. Most participants (14/19) reported being confident in some aspects of their pediatric locomotor exam skills, 3 were not at all confident, and 4 were confident or very confident. Requested topics included: joint examination, assessment of the limping child, assessment of swollen joint(s), investigation of suspected rheumatic disease, and approach to the management of rheumatic diseases.

Of the 12 post-survey respondents, 41.7% attended three virtual education sessions and 58.3% attended four. Half of respondents indicated they had used pGALS since learning it, and half reported they had yet to use it, but that they planned to do so. Participants’ self-reported confidence when examining a child’s locomotor system improved, with 7/12 (58.3%) reporting they were confident in most aspects and 4/12 (33.3%) reporting they were very confident. Overwhelmingly, participants felt more confident in diagnosing and treating the conditions covered (91.7% for each criterion.) Key takeaways included diagnosing rheumatic conditions without expensive laboratory tests and increasing the frequency with which rheumatic conditions should be included in differential diagnosis.

Our educational model of virtual, interactive, brief, synchronous sessions appears to be a cost-effective, high-impact intervention to increase physician exposure to and comfort with pediatric rheumatic conditions worldwide.

## Data Availability

The datasets used and/or analysed during the current study are available from the corresponding author on reasonable request.
